# Medical heuristics and action-research: professionalism versus science

**DOI:** 10.1186/s12913-020-05888-x

**Published:** 2020-12-09

**Authors:** Jean-Pierre Unger, Ingrid Morales, Pierre De Paepe

**Affiliations:** 1 Department of Public Health, Institute of Tropical Medicine, Nationalestraat 155, B-2000 Antwerp, Belgium; 2 Office de la Naissance et de l’Enfance, French Community of Belgium, Chaussée de Charleroi 95, B-1060 Brussels, Belgium

**Keywords:** Medical knowledge, Health management science, Health systems, Operation research, Action-research

## Abstract

**Background:**

Professional knowledge aims at improving practice. It reduces uncertainty in decision-making, improves effectiveness in action and relevance in evaluation, stimulates reflexivity, and subjects practice to ethical standards. Heuristics is an approach to problem-solving, learning, and discovery employing a practical methodology that, although not optimal, is sufficient for achieving immediate goals. This article identifies the desirable, heuristic particularities of research in professional, medical practice; and it identifies what distinguishes this research from scientific research.

**Main text:**

We examine the limits of biomedical and sociological research to produce professional knowledge. Then, we derive the heuristic characteristics of professional research from a meta-analysis of two action-research projects aimed at securing access to essential generic drugs in Senegal and improving physicians’ self-assessment and healthcare coordination in Belgium.

To study healthcare, biomedical sciences ignore how clinical decisions are implemented. Decisions are built into an articulated knowledge system, such as (clinical) epidemiology, where those studied are standardisable - while taking care of patients is an idiosyncratic, value-based, person-to-person process that largely eludes probabilistic methodologies. Social sciences also reach their limits here because descriptive, interpretative methods cannot help with gesture and speech quality, while the management of the patient’s suffering and risks makes each of them unique. Research into medical professionalism is normative as it is intended to formulate recommendations. Scientific data and descriptions are useful to the practitioner randomly, only from the similarities in the environment of the authors and their readers. Such recommendations can be conceived of as strategies, i.e., multi-resource and multi-stage action models to improve clinical and public health practice. Action learning and action-research are needed to design and implement these strategies, because their complexity implies trial and error. To validate a strategy, repeated experiences are needed. Its reproducibility assumes the description of the context. To participate in medical action-research, the investigator needs professional proficiency - a frequent difficulty in academic settings.

**Conclusion:**

Some criteria to assess the relevance of publicly funded clinical and public health research can be derived from the difference between scientific and professional knowledge, i.e. the knowledge gained with real-life experience in the field.

## Background

Cochrane, the founder of Evidence-Based Medicine, considered that when a physician made a clinical decision, he ought to regard the values of the patient and his experience as much as the scientific evidence [1]. This is why medical professionalism is more than a science. It is made up of values, points of view, justifications and gestures, actions, and skills. It is thus a culture.

Clinical, as well as public health knowledge is complex, because medical practice is an art that combines (manual, behavioural and communication) skills, emotions, science, techniques, self-reflection, and philosophical reflection. A large array of disciplines is available to enrich the medical professional culture, but not all of them belong to the medical field. For instance, ethical standards in clinical decision-making call for moral philosophy; and biopsychosocial aspects of health care delivery benefit from psychological sciences.

Professional knowledge ought to be viewed as knowledge that identifies how the subject of study can and ought to be improved under every day conditions. Research into medical professionalism ought to aim to produce knowledge that reduces physicians’^1^ and professionals’ uncertainty in their decision-making, that improves the effectiveness of their actions, that increases relevance in evaluation, and that stimulates reflexivity and ethical behaviour.

To produce knowledge relevant to clinical and public health practice requires heuristic methods. If the Oxford English Dictionary defines them as methods “enabling a person to discover or learn something for themselves: a ‘hands-on’ or interactive heuristic approach to learning”, the more current use defines “heuristics” as “any approach to problem solving, learning, or discovery that employs a practical methodology not guaranteed to be optimal or perfect, but sufficient for the immediate goals.” [2].

Physicians often employ heuristics unknowingly in the interpretation of clinical guidelines, for ethical guidance, or to steer the intangible, symbolic motivation of colleagues and students. In public health, heuristics has been explored with respect to inductive versus deductive reasoning [3], conditions of its critical use [4], techniques for stimulating creativity in seeking health solutions [5], and better representing complex health systems [6, 7].

This article discusses the desirable heuristic particularities of the research aimed at developing professional knowledge in clinical medicine, public health, and healthcare management and the differences that distinguish medical from scientific research using the meta-analysis of two research projects conducted in Belgium and Senegal.

## Main text

K. Lewin described “action-research” as “comparative research on the conditions and effects of various forms of social action and research leading to social action” that uses “a spiral of steps, each of which is composed of a circle of planning, action and fact-finding about the result of the action” [8]. In health, action-research mobilises both professional and social actors.

We derive the heuristic characteristics of research applied to medical professionalism from the meta-analysis of two action-research projects aimed at securing access to essential, generic drugs in Senegal [9] and improving physicians’ self-assessment and healthcare coordination in Belgium [10–14], respectively. We examine their commonalities and elaborate on their objectives, methods, results, and the professionals’ motivations to engage in research involving care and organisational changes.

## Formulating strategies and theories for clinical and public health practice

### How to secure universal access to essential generic drugs? A case study in Senegal

In the 1980s, the Senegalese had limited access to care due to under-financing of public services while private services were absent from peri-urban and rural areas. The national policy focus was still on comprehensive care rather than essential packages, as is the case today. The system featured a substantial rural–urban and rich–poor gap in the availability of services and low levels of public expenditure on health per capita. The health system was not yet characterised by an over-riding pattern of donor assistance and the degree of public sector bureaucracy was not marked, because it did not exclusively focus on disease control, as is the case today. Structural Adjustment Programmes encouraged an internal and international exodus of medical staff [15] and international migration [16]. Allegedly to reduce public expenditure on health, the World Bank promoted a management/property split (public property, private management) in public services granting financial autonomy to university teaching and regional hospitals and to first-line services. These actions paved the way for their privatisation.

By that time, in order to counter the commercialisation of care, and to pilot the strengthening of health systems throughout Western Africa, WHO funded a national project in Senegal called “Primary Health Care” designed to provide technical assistance to district management teams, health centres, and public hospitals. It aimed to integrate health services; to have them co-managed between representatives of users, professionals and the state; and to promote a bio-psychosocial delivery of care. The WHO-funded 1985–88 project strove to improve the population’s access to professional healthcare and to study health development strategies suitable for non-profit health services (including several faith-based health centres). A research and training centre was created in Thiès to improve the government medical officers’ abilities to manage the country’s forty health districts. Its activities connected the following [17]: continuing medical education; operations research to reveal key problems in the participating services; coaching to provide on-site technical and psychological support to district medical teams [18]; action-research to test solutions to prevailing health system deficiencies; health care service planning; disease control; and, *later,* support to central MOH staff to enhance consistency between central planning and field realities.

At that time, austerity policies forced public services to slash their drugs and medical equipment purchases throughout Sub-Saharan Africa, albeit denying access to essential generic drugs at the same time. This reduced access to health care dramatically. The aim of this nested research led by the Primary Health Care project was to formulate advice and managerial tools to secure universal access to safely-prescribed essential generic drugs in defined territories in Senegal.

In the 1980s and 1990s, WHO and UNICEF sought, via the Bamako Initiative [19], to secure access to essential generic drugs in publicly-oriented health centres and general hospitals and to democratise their management in thirty-five LMICs (low- and middle-income countries). The Bamako Initiative financed revolving funds to purchase drugs, contingent on the co-management of public services with users’ representatives. This initiative multiplied the utilisation ratio of first-line services in Guinea and Benin three- and fivefold, respectively [20].

Admittedly, the Bamako Initiative failed in several other countries, because the difficulty of coordinating financial, pharmaceutical and clinical management clashed with nepotism. Indeed, cronyism had the effect of appointing incompetent managers in the health system whilst the management of the Bamako initiative was particularly complex.

The challenge of the research in Senegal was thus to achieve the Bamako Initiative objectives using local resources only. The strategy tested in Kolda (1987 population: 184,000) combined financial, pharmaceutical, and clinical interventions to secure patient access to all essential generic drugs in the first-line public services and referral hospital [9]. Increased population-based health service utilisation rates and pharmaceutical and financial indicators were to measure the strategy’s achievements. Knowledge was the main external input.

With no financial input – only technical assistance – from cooperation agencies, the research team reorganised government health centres one by one and turned them into community-monitored, co-managed structures. A health committee with users’ representatives was established in each health centre (1 per 10,000 pop.) to choose tariffs and payment modes (here, the fee-for-sickness episode was chosen); to define exemptions on social grounds; and to monitor and oversee civil servants, resources, and activities (e.g., by triangulating production data i.e. by comparing two data sources). The committees also set priorities once the accounting break-even point had been reached. For initial financing, the researchers reached revolving funds by organising socio-cultural events.

The first health centre to be “reset” was nested in the district hospital in order to facilitate the design of the pharmaceutical management system. To avoid running out of medicines, the researchers coordinated rationalisation of clinical care with the purchase of pharmaceuticals. After 2 years, universal access to drugs remained a long-term perspective in Kolda district (the inhabitants were far from having all access to all necessary essential and generic medicines), but a publicly oriented drug distribution system had been established. Access to drugs was improved and prescriptions for drugs from private outlets were significantly reduced – another issue with the Bamako Initiative. Access to health care, measured by population-based health service utilisation rates, was multiplied 4.5 times in less than a year.

While the present paper examines its heuristic lessons, some immediate, managerial messages were learned from the experience:

- To monitor access to medicines in countries where they are sorely lacking, governments should, not only monitor their purchase and delivery, but also the use of health services (expressed, for example, by the number of new cases per year and per capita in first-line services and by hospital admissions rates calculated on a population basis).
- A multi-function, systemic planning structure coordinating the hospital and health centres over a territory proved useful to transfer the provision of drugs, of knowledge, and of funds between facilities.
- Financial and pharmaceutical management and clinical training had to be closely coordinated if patients’ healthcare expenditures were to be lowered.
- Professional coaching of district health services by a physician was necessary to coordinate the strategy’s implementation.
- The integration of pharmaceutical stocks in dispensaries and hospitals, as opposed to the separate sale of medicines [21], made it possible to adopt a payment-per-sickness episode system that was conducive to care accessibility and continuity much more than a fee-for-service scheme whilst reducing the unnecessary consumption of both health care and pharmaceuticals.
- Reorganising the hospital pharmacy and rationalising its clinical practice before revamping the health centres proved to be a reasonable option.

### How to improve professionals’ reflexivity and care coordination in high-income countries: a case study in Belgian health services

From the post-war period on, Belgium’s health services have been characterised by self-employed GPs, private non-profit hospitals, weak mid-level managerial structures, and strongly dominant publicly-oriented health financing (Bismarck-style social insurance) [22]. The country’s health system was fragmented, but not highly segmented.

**Table:** 

Health systems are “segmented” when different health subsystems exist for different social classes. Neoliberal policies segment health systems whilst contracting out medical services, separating purchasers and providers, and privatising health services. Consequently, poor patients do not have sufficient access to high-complexity hospitals and technology, a situation aggravated by socio-cultural factors. Health services are said to be “fragmented” when specialists and GPs do not communicate with each other enough about common patients and do not coordinate their clinical decisions, their clinical functions are not defined to cover all health situations, and the various tiers’ functions are not specific to them.	

From 2000 on, the Belgian government used disease-specific programmes to fix deficiencies in clinical coordination, with limited success and commercial insurances and health services began to expand.

This second case study relates to a programme to strengthen professionals’ abilities to assess themselves and to improve clinical coordination beyond institutional boundaries in order to “defragment” the Belgian health system.

As early as 1994, our academic unit launched an in-service strategy called Local Health System (Sylos/Silog in French and Dutch). During a conference, the researchers shared the experiences of African health districts with eighty GPs and specialists and discussed how the underlying concept could help to defragment the Belgian health system “from within”. Fifteen to twenty GPs and specialists sharing common patients volunteered to join a “shadow” executive team to identify and fix issues in the clinical coordination between first-line services and hospital and to detect and correct medical errors, iatrogenic harm, organisational inefficiency, and disease control issues [23, 24]. With coaching by our academic unit and no funding, the volunteers gathered monthly to solve problems and conflicts concerning their shared patients. Case management was reviewed to identify necessary initiatives in a series of areas: teamwork, continuing medical education, health information systems, inter-personal communication, clinical decision-making, professional ethics, medical audits, and the decentralisation of medical techniques from hospitals to GPs. Over the next two decades, several shadow districts joined the initiative in Brussels, Antwerp, Malmedy, and Liège. The project remained unfunded, with one brief exception.

Here follow a few examples of results:

- Negotiations between general practitioners and neurologists made it possible to determine the criteria for managing patients with Alzheimer’s disease between health care levels of the Belgian system.
- Discussions with specialists facilitated the development and dissemination of an algorithm for GP management of patients addicted to heroin.
- GPs have been trained in the use of subcutaneous drips to facilitate home hospitalisation.
- General practitioners’ access to the computerised hospitalisation records has been facilitated.
- Some hospital radiology laboratories and departments have been reorganised to facilitate communication with general practitioners and the results have been modified to allow them to be oriented towards clinical decision-making.
- The average length of hospital stays in surgery has been reduced by delegating preoperative examinations and postoperative follow-up to general practitioners.
- Pain management has been standardised across the areas served by the participating hospitals.
- Training sessions for participating GPs were organised to improve their manual skills in areas as varied as pneumology (spirometry, peak flow, saturometry) or cardiology (reading ECG, mistakes of automatic diagnosis, demand of opinion for difficult ECG, cardio-respiratory reanimation).

What lessons were learnt?

- A small team of motivated professionals working outside the administrative framework can improve quality of care, clinical coordination and medical professionalism over territories.
- The project inspired participating physicians, many of whom had collaborated for more than twenty years, by opening up opportunities for improving the organisation of health care, coordinating care between GPs and hospital specialists, and connecting clinical self-analysis with care management.
- Internal clinical audits designed to identify managerial priorities proved indispensable to represent the patient’s interests whilst preventing physicians being judged on performance outside their teams [23].
- External professional coaches were needed during the first years to support team activities.

### Lessons for research to improve medical professionalism

#### Characteristics of professional practice and knowledge

As per Evidence-Based Medicine (EBM), translating scientific laws and theories into clinical and public health practice requires integrating the clinician’s experience, the patient’s values, and the best available scientific information. Our two studies reveal a third dimension whereby professional knowledge outstrips scientific knowledge.

Managers are traditionally defined as persons entrusted with decision-making aimed at achieving their institutions’ predetermined goals most efficiently. However, our studies show that doctors ought to build and lead teamwork, reflect on practice, coach, educate, train, improve the organisation of health services, manage drugs, coordinate and evaluate healthcare, and contribute to disease and health risk control alongside their clinical duties possibly without their institution’s support, in order to optimise their impacts on health. The two Belgian and Senegalese studies show a) that doctors can significantly improve quality of care and accessibility with clinical and non-clinical, medical interventions; and b) research into medical professionalism may enhance the latter alongside clinical decision-making.

Medical practice and healthcare management cannot be inspired by industrial management, because care delivery is “customised”, and because medical ethics, biopsychosocial decision-making, professional communication, relationships, manual skills, and professionals’ reflectivity are not, or not much, amenable to probabilistic research and qualitative research. Bespoke care delivery and management make heuristics important to research into clinical and public health practice because they evade quantitative probabilistic and qualitative interpretative studies.

#### Contribution of scientific research to professional knowledge

The goal of scientific research is to discover laws and postulate theories that can explain natural or social phenomena, or in other words, build scientific knowledge. Scientific studies ought to be planned systematically before being performed, because scientific methodologies involve making hypotheses and carrying out experiments or empirical observations based on predictions derived from the hypotheses.

Probabilistic quantitative sciences help evaluate drugs and medical equipment, prepare pharmaceutical discovery, and assess disease frequency, symptoms and test results. Qualitative interpretative sciences help describe and explain medical practice. But in general, neither science is able to produce knowledge to improve professional practice.

Although quantification was used to monitor inputs and outputs in the Senegalese experience, scientific methods could not have brought much information relevant to medical practice in the two studied research projects [25] This, because decisions required ethics, rules of thumb, educated guesses, intuitive judgments, common sense, reflection, and philosophical thinking. Physicians were considered “ethical craftsmen”, not just technicians on parts of a production line, as often envisioned by industrial management theory: The concepts and techniques of quantitative analysis were not compatible with the *sufficient* freedom needed by these professionals to provide quality care and even drugs, since the list of drugs available in Kolda had to be negotiated with the prescribers.

Scientific research is unsuitable for the discovery of professional knowledge. If it is quantitative in essence, it will not represent values, personal experience, and skills in a way that is simple to allow validation. If it is qualitative and interpretative, it may uncover the hidden agenda of the institution that determines medical practice but risks ignoring the effectiveness of gesture and speech, as well as the intangible motivation of the caregiver.

With exceptions (such as the McMaster University group) [26], traditional clinical and public health research has usually been normative only if the effects of interventions could be quantified probabilistically, as in the case of disease control and clinical epidemiology. Our two studies show that, as managerial objects, medical professionalism calls for complex interventions that integrate clinical medicine, healthcare management and public health practice, as well as concern for multiple goals, resources, and constraints. By contrast, the vast public health literature on complexity is rarely geared to decision-making, because it raises problems of concept validation and limited reproducibility of experiences.

#### Research into medical professionalism and its heuristic particularities

In medicine, professionalism and scientific excellence are mutually enriching: the former gives the latter the relevance of concerns and scientific excellence contributes to the reflexivity of professional practice and the integration of knowledge.

To theorise advice for “manager-physicians”, researchers relied on a succession of hypotheses that were discarded for better ones (“progressive discovery”). For example, the researchers imagined being able to extricate themselves from the supervision of the Belgian experiment much earlier than they actually did, but the needs for training in public health and sometimes the intricacy of financial tensions between GPs and specialists prevented them doing so. And in Senegal, researchers thought to start the experience by strengthening pharmaceutical supplies for health centres before that of the hospital. Implementation issues could not all be imagined or foreseen at the planning stage. They assessed, through trial and error, the intended and unintended consequences of managerial decisions. Unlike pharmacological research, it is therefore not possible to plan in advance research into medical professionalism and fully control its environment. This is what gives action-research its importance in the discovery of professional medical knowledge.

Action-research is probably the best-known heuristic, praxis-based research methodology. R. Loewenson and co-workers characterise participatory action-research as follows: “Firstly...Participatory action-research aims to overcome the separation between subject and object. Those affected by the problem are the primary source of information and the primary actors in generating, validating and using the knowledge for action. … Secondly, it involves developing, implementing, and reflecting on actions as part of the research and knowledge generation process. Participatory action-research seeks to understand and improve the world by changing it, but does so in a manner that those affected by problems collectively act and produce change as a means to new knowledge” [27].

Both studies were structured as action-research, which others have advocated as a strategy to improve healthcare services and systems [28–30]. As such, their values had to be made explicit, because they are to professional action what standards (effectiveness, efficiency, precision, absence of bias, etc.) are to scientific research. The two studies treated access to professional care as a universal human right. Alongside the Hippocratic Oath’s “self-effacement tenet” (“Into whatsoever houses I enter, I will enter to help the sick”), their design relied on three quality of care features, formulated in 1971 by a Belgian medical activist group [31], and corresponding today to WONCA Europe’s definitions, i.e., holistic modelling, care coordination, and longitudinal continuity of care [32].

Inspired by other teams’ previous efforts along such lines [30, 33, 34], these action-research experiences also relied on explicit publicly-oriented standards for the management and planning of services and systems with a social and professional mission and for disease control programmes. These norms stated in particular that healthcare management should promote the negotiation of decisions amongst professionals, users, mutual aid societies, unions, and the State; and help de-segment and de-fragment health systems.

The conditions under which action-research by medical professionals can improve clinical and public health practice and develop medical knowledge distinguish it from participatory action-research:

- First, for the sake of relevance, health care strategy design and implementation require researchers to learn professional skills and concepts continuously and to be exposed to professional teaching methods, e.g., coaching, technical and psychological supervision, teamwork, audits, demonstrations, observations, in-service continuing medical education, debates [35], and audits. This is why every action-research process involves professional learning.
- Second, researcher-actors need to practice, reflect, and have sufficient professional experience to develop theories relevant to doctors, respond to their problems, and introduce change in clinical and public health practices. In Senegal, for instance, they had to be physicians in order to adjust the clinical decisions of health centre nurses to the available drugs; and in Belgium, to derive care coordination and continuous medical education initiatives from clinical audits and critical incidents. In both studies, they also had to be experienced advisors, as they had to be credible to discuss the problems and solutions with those participating. Therefore, researchers and members of the external advisory team have to be healthcare professionals themselves - which has consequences for academia: practitioner physicians are needed to research medical professional knowledge.

#### Professional knowledge validation

Kant’s categorical imperative states, “Act only according to that maxim whereby you can, at the same time, will that it ought to become a universal law.” Action-research is known to provide local solutions to managerial problems. However, as the main limitation of action-research is said to be the local validity of its conclusions [27], we discuss if and how the knowledge gained with medical action-research can or cannot be transferred to a variety of contexts.

The concept of strategy enables investigator-actors to abide by the categorical imperative when they conceptualise and validate professional knowledge derived from participatory research because it allows the validity of the normative conclusions to be extended beyond the context where the action research was carried out. In the two studies above, participating professionals designed and tested complex interventions on medical practice, service management, and system organisation. They validated the knowledge gained whilst representing interventions with a model. Models enabled them to validate experiments (and to reproduce them in new settings, see below) and to back viewpoints with indicators, observations, and nested studies. Action models can be called strategies and viewed as a way to structure advice guiding doctors’ analyses, decisions, actions, and evaluations based on specific field needs.

**Table:** 

“Strategies” consist of a set of basic interventions (on professionals, patients, services, diseases and/or populations) that must be implemented and coordinated to achieve set goals. They ought to be defined as necessary and sufficient, so that if one of their components fails or is missing (because of poor implementation, for instance), the whole strategy might fail. Their components can be defined in terms of subsystems. For instance, in Kolda, interventions were medical (to adjust orders to drug availability and clinical decisions in the hospital and health centres), pharmaceutical (to structure the supply system), financial (to make the system sustainable and accessible with appropriate modes of payment), and social (to organise community participation in order to control drug procurement).	

#### Professional experiences reproducibility

Repeated experiments in different settings provided opportunities for further knowledge validation and dissemination. Over the next 20 years, we were able to test strategic variants of the Kolda strategy in Burkina Faso, Ecuador, and Bolivia. Health districts covering populations of 150,000–200,000 for improved access to drugs, but these achievements were generally short-lived (3–5 years) because they weren’t supported by the government policy (except in Ecuador).

Similarly, the managerial techniques explored in Brussels were disseminated in Belgium and the acquired know-how became an input in the participatory research [36, 37] of a European Commission-funded project designed to improve the clinical coordination of GPs and specialists in Colombia, Mexico, Brazil, Uruguay, Chile, and Argentina.

Transferring a strategy to other settings entails defining its conditions of success and failure (its “domain of validity”). These health system, cultural, economic, and/or policy facilitators and obstacles can best be studied with repeated tests. Contextualisation ought also to be studied with interdisciplinary methods and be open to political scientists [38] – yet another sizeable challenge for academia [39].

Reflective processes are associated with risks of complacency, confirmation, and recall biases due to unreliable memory, out-dated experiences, and the difficulty of triangulating information. We tried to reduce the effect of these biases using studies that had been published and explicit quality standards.

Countries rarely have programmes to improve medical professionalism although their public services cannot achieve their social mission without adopting a professional mission as well. A Canadian professional association is one of the few that includes professional teaching and evaluation in the profile of an ideal doctor. CanMeds is a framework that identifies and describes the abilities that physicians require to meet the healthcare needs of the people they serve effectively [40]. Another such exception, the NIVEL Institute in Utrecht [41], makes Dutch GPs a proud example of what professional programmes can achieve nationwide. But in general, professional knowledge is slowly being driven out of academia, because praxis-based methodologies do not appeal to academics as they are not prone to publication and are labour intensive.

## Conclusion

### Criteria for professionally-geared medical action-research

This paper offers social and professional criteria to guide medical health practice research and shows the heuristic importance of action-research. The normative, prescriptive character of professional knowledge ought to be a yardstick in medical research, be it clinical or public health. The production and dissemination of professional knowledge ought to be a priority in government-financed health services, and medical and public health schools. Governments ought to finance non-clinical medical activities sufficiently. Medical and public health schools ought to spell out how they assess the professional proficiency of their staff. Because action is the keystone of professionalism, action-research is the key methodology of medical investigation that aims to support professionalism and the organization of care.

Improving the doctors’ problem-solving capacities proved essential to explain the sustained participation of physicians in unfunded or poorly funded research, even, as was the case in Senegal, despite opportunity costs and harsh living conditions. Both the Belgian and Senegalese participatory research echoed the physicians’ professional identity. In Belgium, it improved continuity of care, connected clinical analysis to service management, facilitated mutual learning between GPs and specialists, improved interdisciplinary cooperation and linked continuing medical education to clinical errors. Having a personal impact on the services organisation and a sense of piloting it in their country made the participants feel empowered.

In Kolda, the research transmitted a professional culture aimed at improving peoples’ access to quality care and medication and reducing families’ catastrophic health expenditures. In Belgium, the conveyed culture was aimed at stimulating care coordination and reflectivity. The physicians’ values, viewpoints, and their validation were diffused by analyses, evaluations, and/or reflective methods. Acquiring a professional culture ought to be rewarded in health services and early on in medical curricula.

Introducing professional objectives in academic institutions requires a cultural change. This carries a cost that universities and governments ought to agree to pay for the sake of promoting the human right to individual, professionally-delivered health care. In the meantime, researchers need strong motivation to uphold their medical commitment and, to paraphrase Socrates’s injunction to educators, to “be able to show the effects of their principles in their own life” [42].

## Data Availability

Data sharing is not applicable to this article as no datasets were generated or analysed during the current study.

## Abbreviations

EBM: Evidence-Based Medicine
GP: General Practitioner
SYLOS/SILOG: Local Health System Project

## Footnotes

1 In many countries medical care is a responsibility of non-physician professionals (nurses, clinical officers, feldschers) or shared with midwives, dentists, physical therapists, and psychologists. This discussion may thus be relevant to other professions.

## References

[1] Sackett DL, Rosenberg W, Gray J, Haynes RB, Richardson WS (1996). Evidence based medicine: what it is and what it isn’t. BMJ..

[2] https://en.wikipedia.org/wiki/Heuristic (Accessed 13 Sept 2020).

[3] Cummings L (2013). Public health reasoning: much more than deduction. Arch Public Health.

[4] Ulrich W, Reynolds M. Critical systems heuristics. In: Reynolds M, Holwell S, editors. Systems approaches to managing change: a practical guide. The Open University. London: Published in Association with Springer-Verlag London Limited; 2010.

[5] O’Grady TP, Malloch K. Innovation leadership. Creating the landscape of Healthcare. Sudbury: Jones and Bartlett Publishers; 2010.

[6] Plsek PE, Greenhalgh T (2001). The challenge of complexity in healthcare. BMJ.

[7] Sicotte C, Champagne F, Contandriopoulos AP (1998). A conceptual framework for the analysis of healthcare Organisations’ performance. Health Serv Manag Res.

[8] Lewin K (1946). Action-research and minority problems. J Soc Issues.

[9] Unger JP, Mbaye AM, Diao M (1990). Finances and drugs at the Core of district health service rehabilitation. A case-study in Senegal: the Kolda District. Health Pol Plan.

[10] Unger JP (2000). Les systèmes locaux de santé, un élément de réponse à la crise du secteur de la santé en Belgique ? Un pré-test à Bruxelles. Santé conjuguée.

[11] Unger JP (2003). The local health systems (LHS) project in Belgium. Presentation at the 11th annual EUPHA meeting. Globalisation and Health in Europe: Harmonising Public Health Practices. 20–22 November 2003, Rome, Italy. Abstract. Eur J Public Health.

[12] Unger JP, et al. e-Letter. Health districts in Western Europe. The Belgian local health systems project. (Learning from developing countries: what are the lessons?). BMJ. 2004; Available at : http://bmj.com/cgi/eletters/328/7443/DC1#59220.

[13] Unger J-P (2004). Les systèmes locaux de santé. Santé Conjuguée.

[14] Belche J-L (2016). Intégration entre ligne de soins, d’un patient à une population. PhD thesis.

[15] Buckley RP, Baker J (2008). IMF policies and health in sub-Saharan Africa.

[16] Voorend K. A Welfare Magnet in the South? Migration and Social Policy in Costa Rica. PhD Thesis. Rotterdam: Erasmus University of Rotterdam; 2016.

[17] Unger J-P, Daveloose P, Bâ A, Toure Sene NN, Mercenier P (1989). Senegal Makes a Move towards the Goals of Alma Ata by Stimulating its Health Districts. World Health Forum.

[18] Unger JP, Ghilbert P, De Paepe P. e-Letter. Continuous medical education with (out) coaching? BMJ. 2004; (http://bmj.com/cgi/eletters/328/7446/999#58480). Accessed 6 May 2004.

[19] UNICEF, Bamako Initiative Management Unit. The Bamako Initiative: Reaching Health Goals through Strengthened Services Delivery. New York: Unicef. Bamako Initiative Management Unit (B.I.M.U.); 1990.

[20] Levy-Bruhl D (1997). The Bamako initiative in Benin and Guinea: improving the effectiveness of primary healthcare. Int J Health Plan Man.

[21] Unger JP, Yada A (1993). Ought to medication be distributed in Africa by health services and/or pharmacies? A preliminary evaluation of the Boulgou project in Burkina Faso. Health Pol Plan..

[22] Gerkens S, Merkur S (2010). Belgium: Health system review. Health Syst Trans.

[23] Unger J-P, Marchal B, Dugas S, Wuidar MJ, Burdet D, Leemans P, Unger J (2004). Interface flow process audit: using the patient’s career as a tracer of quality of care and of system organisation. Int J Integr Care.

[24] Belche J-L (2016). Intégration entre lignes de soins : d’un patient à une population. PhD thesis, Liège University.

[25] Unger JP, Morales I, De Paepe P, & Roland M. Integrating medical and public health knowledge - in support of joint medical practice. Forthcoming as part of BMC Health Services Research Volume 20 Supplement 2, 2020: “*The Physician and Professionalism Today: Challenges to and strategies for ethical professional medical practice.*" The full contents of the supplement are available online at https://bmchealthservres.biomedcentral.com/articles/supplements/volume-20-supplement-2. 10.1186/s12913-020-05884-1 PMC7723462 33292178

[26] https://www.mcmasterhealthforum.org Accessed 13 Sept 2020.

[27] Loewenson R, Laurell AC, Hogstedt C, D’Ambruoso L, Shroff Z (2014). Participatory action-research in health systems: a methods reader, TARSC, AHPSR, WHO, IDRC Canada, EQUINET, Harare.

[28] Lallé B. Production de la connaissance et de l’action en science de gestion. Le statut expérimenté de “chercheur–acteur “. Available at : https://www.cairn.info/revue-francaise-de-gestion-2004-1-page-45.htm Accessed 13 Sept 2020.

[29] Lehmann U, Gilson L (2015). Action learning for health system governance: the reward and challenge of co-production. Health Policy Plan.

[30] Grodos D, Mercenier P (2000). Health systems research: a clearer methodology for more effective action. Stud Health Serv Org Policy.

[31] Pour une politique de la santé (1971). Groupe d’étude pour une réforme de la médecine.

[32] Mola E, Eiksson T, Ortiz Bueno MJ, et al. The European definition of general practice/family medicine. Short version. European Academy of Teachers in General Practive, Wonca Europe, 2011 edition.

[33] Smith R, Hiatt H, Berwick D (1999). A shared statement of ethical principles for those who shape and give healthcare. A working draft from the Tavistock group. J Nurs Adm.

[34] Giusti D, Criel B, De Bethune X (1997). Viewpoint: public versus private healthcare delivery: beyond the slogans. Health Policy Plan.

[35] Habermas J (1985). The theory of communicative action, volume 1: reason and the rationalisation of society paperback – march 1.

[36] Vázquez ML, Vargas I, Unger JP (2015). Evaluating the effectiveness of care integration strategies in different healthcare systems in Latin America: the EQUITY-LA II quasi experimental study protocol. BMJ Open.

[37] www.equity-la.eu/en/ Accessed 13 Sept 2020.

[38] Hunter D (2015). Role of politics in understanding complex, messy health systems: an essay by David J Hunter. BMJ.

[39] Unger JP (2011). The production of critical theories in Health Systems Research and Education. An epistemological approach to emancipating public research and education from private interests. Health Culture Soc.

[40] 2019 Royal College of Physicians and Surgeons of Canada. http://www.royalcollege.ca/rcsite/canmeds/canmeds-framework-e Accessed 13 Sept 2020.

[41] https://www.nivel.nl/nl/contactgegevens-van-het-nivel Accessed 27 July 2019.

[42] Gros F, Foucault M (2004). Introduction générale. Philosophie. Anthologie. Folio essais, Paris, Gallimard.

